# Supplementary material for: The genome sequence of the Buff Arches, Habrosyne pyritoides (Hufnagel, 1766)
Source: Wellcome Open Res. 2026 Mar 26;8:480. Originally published 2023 Oct 20. [Version 2] doi: 10.12688/wellcomeopenres.20151.2 (PMC13153771; doi:10.12688/wellcomeopenres.20151.2)
Supplement: Peer review history

# Reviewer report: Reviewer response for version 2

**DOI:** 10.21956/wellcomeopenres.28652.r152909

**Published:** 2026-05-07

**Authors:** Li-Wei Wu

**Affiliations:** 1 Tunghai University, Taichung, Taichung City, Taiwan

The article presents a high-quality genome assembly for *Habrosyne pyritoides*, generated using a combination of long-read Pacific Biosciences HiFi sequencing, Hi-C data, and Illumina short reads. The results assembly comprises 30 autosomes and one Z chromosome, consistent with the chromosome number reported for many lepidopteran insects. The result also shows high completeness, with a BUSCO score of 98.9%. Overall, the authors provide a valuable and high-quality genomic resource that will be useful for future comparative genomic, evolutionary, and functional studies.

 One point that may merit clarification concerns the DNA source. Because abdominal tissue was used and the specimens were collected from the field, potential contamination from gut-associated microorganisms could be a concern. It would be helpful if the authors could clarify whether microbial contamination was considered during sample preparation or whether any specific bioinformatic filtering steps were applied to detect and remove possible contaminants during genome assembly and annotation. Overall, this appears to be a well-executed work with a sound methodological workflow. The results are reasonable and provide a useful genomic resource for future research. I look forward to seeing further studies that build upon this genome assembly.

Are sufficient details of methods and materials provided to allow replication by others?

Yes

Is the rationale for creating the dataset(s) clearly described?

Yes

Are the datasets clearly presented in a useable and accessible format?

Yes

Are the protocols appropriate and is the work technically sound?

Yes

Reviewer Expertise:

conservation genetics, phylogenetics

I confirm that I have read this submission and believe that I have an appropriate level of expertise to confirm that it is of an acceptable scientific standard.

# Reviewer report: Reviewer response for version 2

**DOI:** 10.21956/wellcomeopenres.28652.r152094

**Published:** 2026-05-06

**Authors:** Fernando Seixas

**Affiliations:** 1 Wellcome Sanger Institute (Ringgold ID: 47665), Hinxton, England, UK

In the Genome Note, the authors present a chromosome-level genome assembly and annotation for *Habrosyne pyritoides.*

 As a Genome Note, I would expect that it should provide a complete and clear explanation of the molecular and analytical methods used to generate the data, the genome assembly and its annotation. However, I find the manuscript in its current state lacking in this respect. In particular the methodology section lacks sufficient detail; key aspects such as protocols, library preparation, and which technologies or platforms were used are often not adequately described and often different technologies are mentioned in the same paragraph. The manuscript also does not seem to follow the typical structure of other Genome notes, which I found a bit confusing and made it difficult to read. Please find more detailed comments below:

**Genome Sequence Report**

 1. The raw data generated for PacBio and Chromium 10X should be reported – e.g. X Gb (gigabases) from Y million reads. The coverage then depends on the assembly size or estimated haploid genome size (which are only generated downstream). And could you please also specify the amount of HiC data generated? 

 2. “The whole assembly sequence was assigned to 31 chromosomal-level scaffolds, representing 30 autosomes and the Z sex chromosome.”. How was the Z chromosome identified?

 3. “The mitochondrial genome was also assembled and can be found as a contig within the multifasta file of the genome submission.”. What is the size and accession number?

**Methods**

 1. “Protocols for high molecular weight (HMW) DNA extraction [...]”. I believe this sentence should be preceded by a subheader ‘Nucleic acid extraction’. Also, in this section the authors mention that HMW DNA extracted for the abdomen was used for 10X Chromium sequencing and RNA extracted from the head and thorax used for RNA sequencing. However there is no mention of the DNA extractions used for PacBio and Hi-C sequencing.

 2. I believe the section currently labelled as ‘Sequencing’ should be divided into different subsections since different sequencing technologies (Pacbio, Chromium 10X, Hi-C and RNA) were used and each of these required different methodologies for the preparation of the libraries and different sequencing platforms. For instance, in other Genome Notes divide this is found under different sections named‘PacBio HiFi library preparation and sequencing’,  ‘Hi-C’, and ‘RNA library preparation and sequencing’.  I believe that the authors should do the same and describe more thoroughly how the different libraries were prepared, which kits were used and protocols were used, and which platforms and e.g. types of cells were used for sequencing, etc. 

 3. ‘Genome annotation’. Again I find this section lacking information regarding how the gene annotation was performed. For instance, what protein evidence was used for the annotation? And since RNA data was generated for this individual, was it used to help in gene annotation? If not, what was it used for?

Are sufficient details of methods and materials provided to allow replication by others?

No

Is the rationale for creating the dataset(s) clearly described?

Yes

Are the datasets clearly presented in a useable and accessible format?

Partly

Are the protocols appropriate and is the work technically sound?

Yes

Reviewer Expertise:

Evolutionary Biology; Bioinformatics

I confirm that I have read this submission and believe that I have an appropriate level of expertise to state that I do not consider it to be of an acceptable scientific standard, for reasons outlined above.

# Reviewer report: Reviewer response for version 1

**DOI:** 10.21956/wellcomeopenres.22313.r107849

**Published:** 2024-11-06

**Authors:** Hunter K Walt

**Affiliations:** 1 Mississippi State University, Mississippi, Mississippi, USA

In this data note, the authors provide a chromosome-level genome assembly for the Buff arches, a moth found across Europe that has also been reported in Japan. The genome is highly contiguous and has a high completeness score according to appropriate metrics. This high-quality genome is valuable as it can be used for comparative genomic studies in the Lepidoptera and also as a reference for other genomic/transcriptomic analyses in this species. 

 Overall, this data report is well done and the genome is similar to other genomes sequenced within this family. The report is succinctly written in the standard Darwin Tree of Life format. 

 I only have a couple of comments to make.

 1.) MitoHiFi uses a mitogenome from a closely related organism to assemble novel mitochondrial genomes. I understand that it can automatically infer this, however, the species that was used for this assembly should be reported to ensure reproducibility.

 2.) Generally, genomes should be repeat-masked before annotation as it increases accuracy. Was the genome repeat-masked before using the BRAKER2 pipeline? This should be reported to ensure the accuracy and reproducibility of the annotation. Additionally, I suggest reporting some information about the repetitive landscape such as the percent of the genome that was masked.

Are sufficient details of methods and materials provided to allow replication by others?

Partly

Is the rationale for creating the dataset(s) clearly described?

Yes

Are the datasets clearly presented in a useable and accessible format?

Yes

Are the protocols appropriate and is the work technically sound?

Yes

Reviewer Expertise:

Insect genomics and evolution, transcriptomics, metagenomics

I confirm that I have read this submission and believe that I have an appropriate level of expertise to confirm that it is of an acceptable scientific standard.

# Reviewer report: Reviewer response for version 1

**DOI:** 10.21956/wellcomeopenres.22313.r79526

**Published:** 2024-06-10

**Authors:** Ma. Anita Bautista

**Affiliations:** 1 University of the Philippines-Diliman, Quezon City, Philippines

The paper focuses on generating an assembled genome for *Habrosyne pyritoides* (the Buff Arches, Arthropoda, Insecta, Lepidoptera, Drepanidae). The availability of this genome from another species under Drepanidae will enable comparative studies across the Lepidoptera, especially those present in the Atlantic Archipelago of Britain and Ireland.

 While the paper published useful genome information, I found that some details are lacking in the methodology or a few information need to be clarified. For example, RNA was extracted and sequenced for annotation purposes, but can the authors mention if the RNAs were DNAse treated? Also, were there replications needed for RNA Seq? What platforms were used to generate the transcriptome sequences?

 Finally, I found that the writing style used and even how the paper was organized was highly similar to another published paper on another Drepanidae. See **Crowley and Philips, 2023**

 https://wellcomeopenresearch.org/articles/8-458. 

 The paper must be re-written in another style.

Are sufficient details of methods and materials provided to allow replication by others?

No

Is the rationale for creating the dataset(s) clearly described?

Yes

Are the datasets clearly presented in a useable and accessible format?

Yes

Are the protocols appropriate and is the work technically sound?

Yes

Reviewer Expertise:

Insect genomics, transcriptomics, metagenomics

I confirm that I have read this submission and believe that I have an appropriate level of expertise to confirm that it is of an acceptable scientific standard, however I have significant reservations, as outlined above.

## References

[1] : The genome sequence of the Yellow Horned, Achlya flavicornis (Linnaeus, 1758). *Wellcome Open Research* .2023;8: 10.12688/wellcomeopenres.19846.1 10.12688/wellcomeopenres.19846.1 PMC1185100340008385

# Response

**Published:** 2026-02-04

**Authors:** Tree of Life Team Sanger

**Affiliations:** Wellcome Trust Sanger Institute, UK

We thank the reviewer for their comments on this data note. The similarity in structure and writing style to other published data notes, including Crowley and Phillips (2023), is intentional and reflects the standardised format used for genome notes generated as part of the Darwin Tree of Life project. These articles follow a common structure to ensure consistency and comparability across a large number of species, and to allow readers to locate key information efficiently. This approach is aligned with the scope and aims of the project, which is producing genome notes for many thousands of taxa using a consistent analytical and reporting framework. We have included links to the appropriate wet lab protocols used in the Methods section, and to the github page for production code in the Data availability section. Tree of Life team, Wellcome Sanger Institute
